# Two-Dimensional-PAGE Coupled with nLC-MS/MS-Based Identification of Differentially Expressed Proteins and Tumorigenic Pathways in MCF7 Breast Cancer Cells Transfected for JTB Protein Silencing

**DOI:** 10.3390/molecules28227501

**Published:** 2023-11-09

**Authors:** Madhuri Jayathirtha, Taniya Jayaweera, Danielle Whitham, Isabelle Sullivan, Brîndușa Alina Petre, Costel C. Darie, Anca-Narcisa Neagu

**Affiliations:** 1 Biochemistry & Proteomics Laboratories, Department of Chemistry and Biomolecular Science, Clarkson University, 8 Clarkson Avenue, Potsdam, NY 13699, USA; jayathm@clarkson.edu (M.J.); jayawetm@clarkson.edu (T.J.); whithad@clarkson.edu (D.W.); sullivil@clarkson.edu (I.S.); cdarie@clarkson.edu (C.C.D.); 2 Laboratory of Biochemistry, Department of Chemistry, “Alexandru Ioan Cuza” University of Iasi, Carol I bvd, No. 11, 700506 Iasi, Romania; 3 Center for Fundamental Research and Experimental Development in Translation Medicine–TRANSCEND, Regional Institute of Oncology, 700483 Iasi, Romania; 4 Laboratory of Animal Histology, Faculty of Biology, “Alexandru Ioan Cuza” University of Iasi, Carol I Bvd. No. 22, 700505 Iasi, Romania

**Keywords:** breast cancer (BC), MCF7, JTB protein silencing, overexpressed JTB interactome, downregulated JTB interactome, tumorigenic pathways

## Abstract

The identification of new cancer-associated genes/proteins, the characterization of their expression variation, the interactomics-based assessment of differentially expressed genes/proteins (DEGs/DEPs), and understanding the tumorigenic pathways and biological processes involved in BC genesis and progression are necessary and possible by the rapid and recent advances in bioinformatics and molecular profiling strategies. Taking into account the opinion of other authors, as well as based on our own team’s in vitro studies, we suggest that the human jumping translocation breakpoint (hJTB) protein might be considered as a tumor biomarker for BC and should be studied as a target for BC therapy. In this study, we identify DEPs, carcinogenic pathways, and biological processes associated with JTB silencing, using 2D-PAGE coupled with nano-liquid chromatography tandem mass spectrometry (nLC-MS/MS) proteomics applied to a MCF7 breast cancer cell line, for complementing and completing our previous results based on SDS-PAGE, as well as in-solution proteomics of MCF7 cells transfected for JTB downregulation. The functions of significant DEPs are analyzed using GSEA and KEGG analyses. Almost all DEPs exert pro-tumorigenic effects in the JTB^low^ condition, sustaining the tumor suppressive function of JTB. Thus, the identified DEPs are involved in several signaling and metabolic pathways that play pro-tumorigenic roles: EMT, ERK/MAPK, PI3K/AKT, Wnt/β-catenin, mTOR, C-MYC, NF-κB, IFN-γ and IFN-α responses, UPR, and glycolysis/gluconeogenesis. These pathways sustain cancer cell growth, adhesion, survival, proliferation, invasion, metastasis, resistance to apoptosis, tight junctions and cytoskeleton reorganization, the maintenance of stemness, metabolic reprogramming, survival in a hostile environment, and sustain a poor clinical outcome. In conclusion, JTB silencing might increase the neoplastic phenotype and behavior of the MCF7 BC cell line. The data is available via ProteomeXchange with the identifier PXD046265.

## 1. Introduction

Discovering and validating novel biomarkers, especially for early cancer diagnosis, as well as molecular targets for advanced therapies in breast cancer (BC), necessitate the handling of accurate gene expression datasets [1]. The identification of new cancer-associated regulatory genes/proteins, the characterization of their expression variations, the interactomics-based assessment of differentially expressed genes/proteins (DEGs/DEPs), and understanding the tumorigenic pathways and biological processes involved in BC genesis and progression are possible by the rapid and recent advances in bioinformatics and molecular profiling strategies or analytical techniques, especially based on high-throughput sequencing and mass spectrometry (MS) developments.

In 1999, Hatakeyama et al. reported the human jumping translocation breakpoint (hJTB) as a novel transmembrane protein gene at locus 1q21, a region called the epidermal differentiation complex (EDC), involved in unbalanced jumping translocation, suggesting the JTB’s association with tumor progression [2]. Moreover, Tyszkiewicz et al. (2014) showed that the EDC molecules were involved in important mechanisms in adenocarcinomas [3], while other authors showed that chromosomal translocations were a hallmark for cancer [4], jumping translocations (JTs) being usually identified in tumors [5]. In 2007, Kanome et al. stated that JTB is a transmembrane protein with an unknown function; however, the authors observed that JTB expression was suppressed in many tumor types, emphasizing its role in the malignant transformation of cells [6]. Platica et al. (2000) showed that hJTB cDNA had a 100% homology with prostate androgen-regulated (PAR) gene isolated from an androgen-resistant prostate cancer cell line [7]. The same authors reported that PAR/JTB expression was upregulated in all studied prostatic carcinoma cell lines compared with normal prostatic tissue, in androgen-resistant prostate cancer cell lines in comparison with androgen-sensitive prostate cells, in MCF7 and T47D BC cell lines, as well as in all the primary breast tumors studied compared to their normal counterparts. Moreover, Platica et al. (2011) observed that the downregulation of PAR levels in DU145 cells resulted in defects in centrosome segregation, failed cytokinesis and chromosome alignment, and an increased number of apoptotic cells, polyploidy, and aberrant mitosis that could lead to genomic instability and tumorigenesis [8]. These authors suggested that the PAR overexpression in several human cancers might be a putative target for therapy. Pan et al. (2009) showed that JTB may play a critical role in liver carcinogenesis [9]. Functionally, JTB has been reported as a regulator of mitochondrial function, cell growth, cell death and apoptosis, as well as being a protein involved in cytokinesis/cell cycle activities [6,8].

MCF7 is a middle aggressive and non-invasive BC cell line that has been used for membrane protein enrichment proteomic analyses [10] as well as for the identification of dysregulated signaling pathways and cellular targets of different compounds with anti-tumorigenic activity [11]. We also show that the upregulated expression of DEPs in the JTB^low^ condition, investigated by SDS-PAGE followed by nLC-MS/MS proteomics in a transfected MCF7 BC cell line, promotes cancer cell viability, motility, proliferation, invasion, the ability to survive in hostile environments, metabolic reprogramming, and the escaping of tumor cells from host immune control, leading to a more invasive phenotype for MCF7 cells. Several downregulated DEPs in a low-JTB condition also promote the invasive phenotype of MCF7 cells, sustaining cell proliferation, migration, invasion, and tumorigenesis [12]. Several DEPs identified during JTB silencing by in-solution digestion followed by nLC-MS/MS that were complementary to the initial in-gel based ones [12], especially upregulated proteins, are known to emphasize pro-tumorigenic activities in a downregulated state [13].

Taking into account the previously cited references [2,6,7,8,9], as well as based on our own team’s studies [12,13,14,15], we suggest that the JTB protein might be a tumor biomarker for BC and should be studied as a target for cancer therapy. In this study, we identify the DEPs and carcinogenic pathways associated with JTB silencing, using 2D-PAGE coupled with nano-liquid chromatography-tandem mass spectrometry (nLC-MS/MS) proteomics applied to the MCF7 breast cancer cell line, for complementing and completing our previous results based on SDS-PAGE [12], as well as the in-solution proteomics of MCF7 cells transfected for JTB downregulation [13]. We concluded that almost all DEPs exert pro-tumorigenic effects in JTB^low^ conditions, sustaining the tumor suppressive function of JTB. The function of DEPs has been analyzed using GSEA and KEGG, while STRING analysis has been applied to construct the protein-protein interaction network of the JTB^low^-related proteins that exert a PT activity. The identified DEPs are involved in several signaling and metabolic pathways and biological processes that exert pro-tumorigenic (PT) roles: EMT, tight junction, cytoskeleton organization, ERK/MAPK, PI3K/AKT, Wnt/β-catenin, mTOR, c-MYC, NF-κB, IFN-γ and IFN-α response, UPR, and metabolic reprogramming.

## 2. Results and Discussion

**Figure 1:**
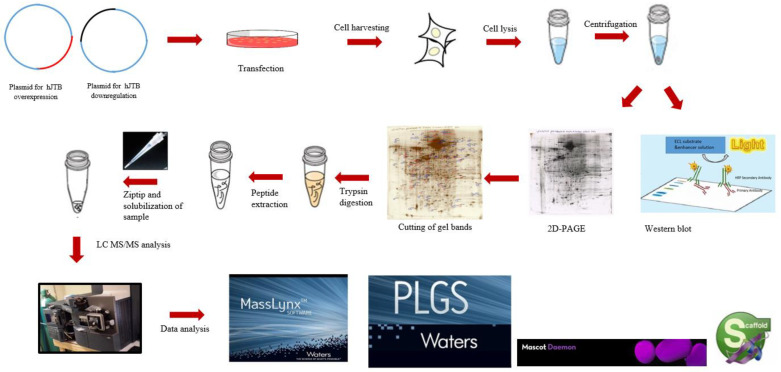
The workflow for cellular proteomics followed by 2D-polyacrylamide gel (2D-PAGE) coupled with nLC-MS/MS analysis of the cell lysates.

Using 2D-PAGE coupled with nLC-MS/MS proteomics, the present study identified 45 significantly dysregulated proteins, 37 upregulated and 8 downregulated, in the MCF7 BC cell line transfected for JTB silencing. The workflow for cellular proteomics followed by 2D-polyacrylamide gel (2D-PAGE) coupled with nLC-MS/MS analysis of the cell lysates is presented in the Figure 1.

**Figure 2:**
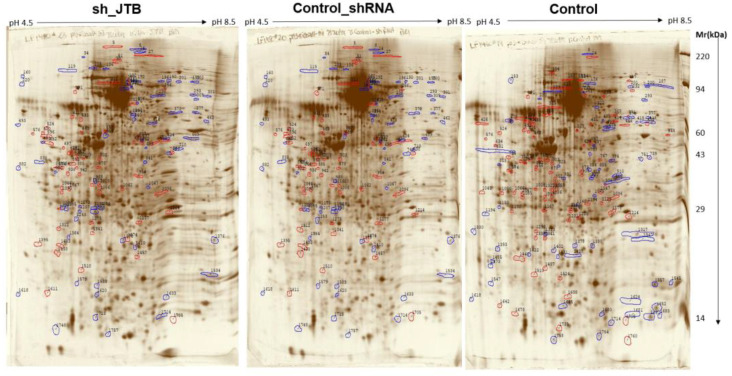
Images of sh_JTB (**left**), control_shRNA (**middle**), control (**right**) silver stained 2D polyacrylamide gels. Polypeptide spots increased in each compared gels (**top** vs. **bottom**) are shown in blue, while spots decreased are outlined in red.

**Figure 3:**
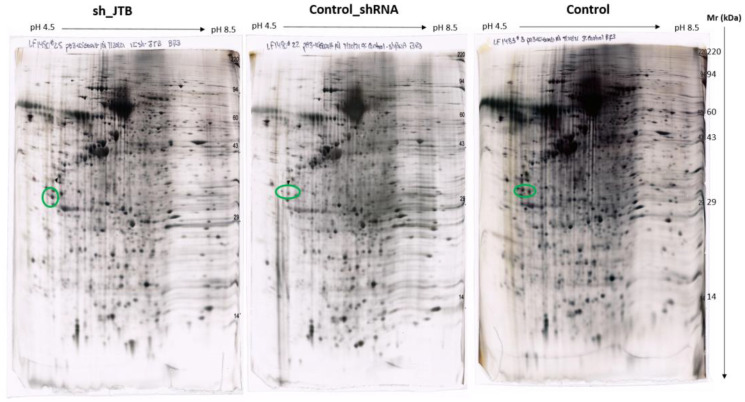
Images of sh_JTB (**left**), control_shRNA (**middle**), control (**right**) silver stained 2D polyacrylamide gels. The circles on each 2D-polyacrylamide gel shows the location of Isoelectric focusing internal standard Tropomyosin of Mw: 33000 and a pI of 5.2.

There were 131 dysregulated spots in the control_shRNA vs. sh_JTB and 153 differences in the control vs. sh_JTB (Figure 2 and Figure 3). A total of 284 spots were selected for Nano LC- MS/MS analysis, as previously described [16].

We only analyzed proteins that have a protein score of above 40 and *p*-value < 0.05. HSPB1, HSPA4, MCM6, ACTB, ACTN1, PFN2, TUBA1A, EEF2, PCK1, PCK2, GPI, FARSB, GARS1, LARS1, RARS1, PSMC6, CCT2, CCT3, IFIT1, PTBP1, DDX19A, UTY, NCAM2, RHBDD1, CS, LAMTOR3, OGA, ZNF114, PA2G4, SRM, GSTM3, NCKAP1, PRDX3, REB8A, RAB8B, RAB15 and RAB35 are overexpressed, while TUBB4B, CAPN2, ELFN2, SLC9AR1, ANXA4, YWHAZ, YWHAE, and PSMB9 proteins were found to be significantly downregulated. GSEA analysis was performed for the downregulated JTB condition using the H (hallmark gene sets) collection in MSigDB. Analysis of the H collection revealed four upregulated pathways, including proteins important for interferon alpha response (IFN-α), interferon gamma response (IFN-γ), Myc targets V1, and unfolded protein response (UPR). Two downregulated pathways comprised proteins involved in estrogen response late and estrogen response early pathways (Table 1). We also performed Kyoto Encyclopedia of Genes and Genomes (KEGG) analysis and here we emphasized the enriched biological processes in pro-tumorigenic proteins identified in downregulated JTB conditions using GeneCodis website (https://genecodis.genyo.es/, accessed on 22 October 2023). There were 25 upregulated and two downregulated proteins with pro-tumorigenic (PT) potential, which were then submitted for protein-protein interaction (PPI) network construction with Search Tool for the Retrieval of Interacting Genes/Proteins (STRING) database (https://string-db.org/, accessed on 22 October 2023), to analyze the specific interaction network associated with the JTB^low^ condition in the transfected MCF7 BC cell line. A total of 27 nodes and 69 edges were mapped in the PPI network, with an average node degree of 5.11, an average local clustering coefficient of 0.632, and a PPI enrichment *p*-value 6.44 × 10^−12^.

To emphasize the role of the JTB-interactome, we analysed the pro-tumorigenic (PT) and anti-tumorigenic (AT) function of these proteins, as well as the neoplastic dysregulated pathways and biological processes (Table 2).

Analyzing the data from Table 2, we observed that 38 DEPs emphasize a pro-tumorigenic (PT) role and 5 DEPs are known to have anti-tumorigenic (AT) activity in the MCF7 BC cell line transfected for JTB downregulation. 

### 2.1. JTB Silencing Is Associated with Neoplastic Abilities of MCF7 Transfected Cells

The epithelial-mesenchymal transition (EMT) process facilitates the local invasion in cancer. We identified a plethora of upregulated and downregulated DEPs directly or indirectly involved in EMT process: HSPB1, HSPA4, MCM6, ACTN1, PFN2, EEF2, IFIT1, DDX19A, GPI, TUBA1A, CS, PA2G4, GSTM3, NCKAP1, and TUBB4B (Table 2). According to previously published data, HSPB1 and HSPA4 are members of the HSP family that promote EMT in association with the increased invasiveness of cancer cells [13]. Also, the EMT process is subjected to metabolic regulation, while the metabolic pathways adapt to cellular changes during the EMT. The mammalian or mechanistic target of rapamycin (mTOR) pathway becomes aberrant in various types of cancer. The hyperactivation of mTOR signaling pathway promotes cell proliferation and metabolic reprogramming that initiates tumorigenesis and progression [129]. HSPA4, PCK1, PCK2, LARS1, PSMC6, LAMTOR3, SRM, and SLC9AR1 proteins are involved in mTOR pathway activation in MCF7 cells transfected for JTB silencing. The mitogen-activated protein kinase (MAPK) signaling pathway regulates proliferation, differentiation, apoptosis and stress responses, while the overexpression of extracellular signal-regulated kinases ERK1 and ERK2 is critical in cancer development and progression [130]. We identified MCM6, PCK1, LAMTOR3, GSM3, and REB8A as pro-tumorigenic proteins involved in ERK/MAPK signaling pathways in the JTB^low^ condition. Also, the phosphatidylinositol-3-kinase (PI3K/AKT) pathway is one of the most hyper-activated intracellular pathways in many human cancers, contributing to carcinogenesis, cell proliferation, invasion and metastasis [131]. HSPA4, CCT3, PTBP1, and RAB35 are pro-tumorigenic proteins involved in PI3K/AKT pathway. The Wnt/β-catenin signaling pathway facilitates cancer stem cell renewal, cell proliferation and differentiation, being involved in carcinogenesis and therapy response [132]. Several DEPs, such as POTEF/ACTB, PSMC6, CCT3, IFIT1, and RAB8B are dysregulated proteins involved in Wnt/β-catenin signaling pathway, which emphasize pro-tumorigenic activities. NF-κB is an important signaling pathway involved in cancer development and progression, which controls the expression of several target genes and mediates cancer cell proliferation, survival and angiogenesis [133]. Thus, HSPB1/HSP27 and POTEF have been identified as proto-oncogenic proteins involved in this pathway. The unfolded protein response (UPR) is known as a pro-survival mechanism involved in progression of several cancers, such as BC, prostate cancer, and glioblastoma multiforme [134]. Here, JTB silencing was associated with UPR-related proteins, such as EEF2 and IFIT1.

The vesicle transport regulators play key roles in tumor progression, including uncontrolled cell growth, invasion and metastasis [104]. Ras-related proteins, small GTP-binding proteins of the Rab family, are dysregulated in malignant cells, affecting intracellular and membrane traffic, as well as proliferation and metastasis, reducing the survival rate of patients [101]. Ras-related protein Rab-8A (RAB8A) and Ras-related protein Rab-8B (RAB8B) were significantly upregulated in this experiment. Gene ontology enrichment analysis identified the following biological processes enriched in these upregulated proteins: vesicle docking involved in exocytosis, regulation of exocytosis, regulation of protein transport, protein secretion, protein import into peroxisome membrane, Golgi vesicle fusion to target membrane, protein localization to plasma membrane and cell junction organization. RABA8 was reported as overexpressed in BC tissues [101], while RAB8B was upregulated in TC [102]. The RAB8A silencing inhibits the proliferation, migration and invasion of BC cells through suppression of AKT and ERK1/2 phosphorylation [101]. RAB8B is required for the Wnt/β-catenin signaling pathway [103], while its overexpression promotes the activity and internalization by caveolar endocytosis of LRP6, a member of the low-density lipoprotein receptor superfamily of cell-surface receptors, which is involved in cell proliferation, migration, and metastasis [135]. RAB15 is involved in trafficking cargo through the apical recycling endosome (ARE) to mediate transcytosis [104]. It is overexpressed in liver cancer cells [105] and is associated with the susceptibility of cells to DNA damage-induced cell death [105]. RAB35 is an oncogenic protein that enhances the invasion and metastasis of BC cells [107].

**Figure 4:**
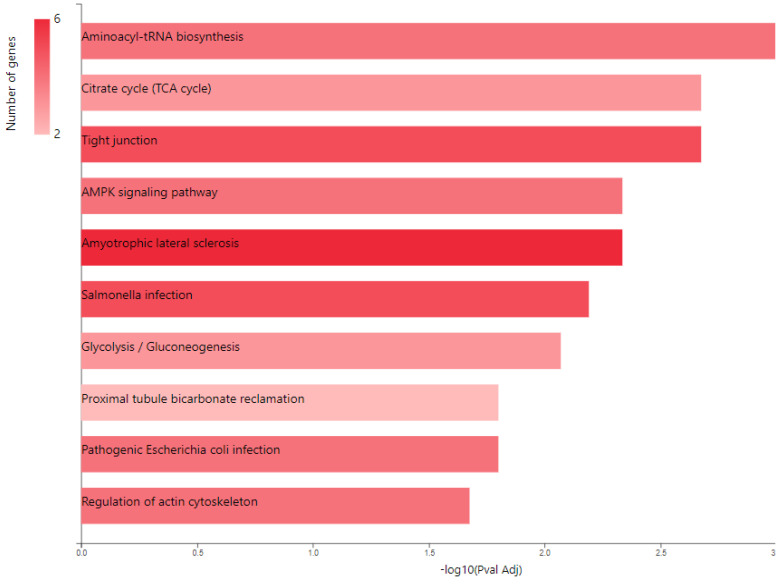

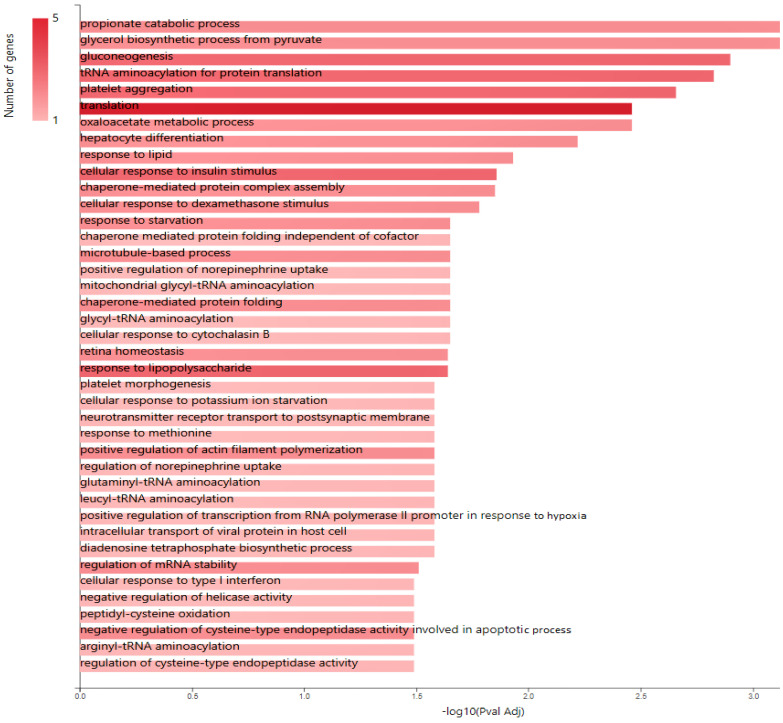
KEGG pathway analysis of pro-tumorigenic (PT) proteins in downregulated JTB condition; **B**. Gene ontology (GO) enrichment analysis of proteins in MCF7 BC cell line transfected for JTB downregulation: biological processes (BP) enriched in PT proteins. The analysis was performed using GeneCodis website (https://genecodis.genyo.es/, accessed on 22 October 2023).

KEGG pathway analysis (Figure 4) also emphasized the following enriched pathways in the MCF7 BC cell line transfected for JTB downregulation: KEGG_Tight junction and KEGG_Regulation of actin cytoskeleton. De Abreu Pereira et al. (2022) showed that highly expressed proteins and biological processes in HCC-1954 (HER2+), a very invasive and metastatic BC cell line, are classified as tight junctions and cytoskeleton proteins, as compared to an MCF7 BC cell line that emphasized proteins related to proteasome and histones in correlation with the higher rate of mutation in MCF7 BC cells [10].

### 2.2. Glucose Metabolism Reprogramming in JTB Downregulated Condition

Multiple cancer cell metabolic pathways are reprogrammed and adapted to sustain cell proliferation, tumor growth, and metastasis in tumor progression, especially under a nutrient deprivation condition. KEGG pathway analysis (Figure 4) emphasized several metabolic enriched pathways in the MCF7 BC cell line transfected for JTB downregulation: TCA cycle (KEGG_Citrate cycle/TCA cycle) and Glycolysis/Gluconeogenesis (KEGG_Glycolysis/Gluconeogenesis), while GO analysis showed as upregulated: gluconeogenesis (GO_BP Gluconeogenesis) and pyruvate metabolism (GO_BP_Glycerol biosynthetic process from pyruvate). The highlighted dysregulation of propionate metabolism (GO_BP Propionate catabolic process) is also known to contribute to a pro-aggressive state in BC cells, increasing cancer cell metastatic ability [136]. Phosphoenolpyruvate (PEP) carboxykinases (PCK1/PEPCK-C, cytosolic isoform, and PCK2/PEPCK-M, mitochondrial isoform) have been shown to be multifunctional enzymes, critical for the growth of certain cancers [137], sustaining cell cycle progression and cell proliferation [39,40]. Thus, in the absence of glucose, cancer cells may synthesize essential metabolites using abbreviated forms of gluconeogenesis, as a reverse phase of glycolysis, especially by PCK1 and PCK2 expression [137], both well known for their key roles in gluconeogenesis and regulation of TCA cycle flux [39]. PCK1 has been reported as a tumor-suppressor in cancers arising from gluconeogenic tissues/organs, such as liver and kidney, while it acts as a tumor promoter in many human cancers arising in non-gluconeogenic tissues [138]. Consequently, PCK1 was reported as an overexpressed oncogene in colon, thyroid, breast, lung, urinary tract, and melanoma cancers [36], while it was found as a downregulated tumor suppressor in tumors arising in gluconeogenic tissues of liver and kidney, such as in HCC [36], and ccRCC [37]. Here we showed that MCF7 cells transfected for JTB downregulation markedly upregulated cytosolic PCK1, which was described as a molecular hub that regulates glycolysis, TCA cycle and gluconeogenesis to increase glycogenesis via gluconeogenesis [139]. PCK1, as a key rate-limiting enzyme in gluconeogenesis, catalyzes the conversion of oxaloacetate (OAA) to PEP (GO_BP oxaloacetate metabolic process) [138] and links the TCA and glycolysis/gluconeogenesis [40]. The expression of PGK1 leads to the biosynthesis of glucose-6-phosphate (G6P) that can be used by different pathways, including conversion to glucose, glycolysis, pentose phosphate pathway (PPP) or glycogenesis [139]. Glucose-6-phosphate isomerase (GPI) that interconverts G6P and fructose-6-phosphate (F6P) is also overexpressed in the downregulated JTB condition (GO_BP Gluconeogenesis). Cytoplasmic GPI is a glycolytic-related enzyme secreted in the extracellular matrix (ECM) of cancer cells, where it is called an autocrine motility factor (AMF) [62], and functions as a cytokine or growth factor [63]. GPI is overexpressed in BC [63], LUAD/NSCLC, glioblastoma, ccRCC [64], and GC [62]. This glycolytic enzyme is involved in cell cycle, cell proliferation, correlates with immune infiltration, cell migration and invasion [64], while silencing suppressed proliferation, migration, invasion, glycolysis, and induced apoptosis (GO_BP Negative regulation of cysteine-type endopeptidase activity involved in apoptotic process) [62]. PCK1 also enhances the PPP, which produces ribose-5-phosphate for nucleotide synthesis and NADPH for biosynthetic pathways [138]. Abundant NADPH ensures high levels of reduced glutathione (GSH) [139], known for its important intracellular antioxidant role, which acts as a regulator of cellular redox state as well as a controller of cell differentiation, proliferation, apoptosis, ferroptosis and immune function [140].

**Figure 5:**
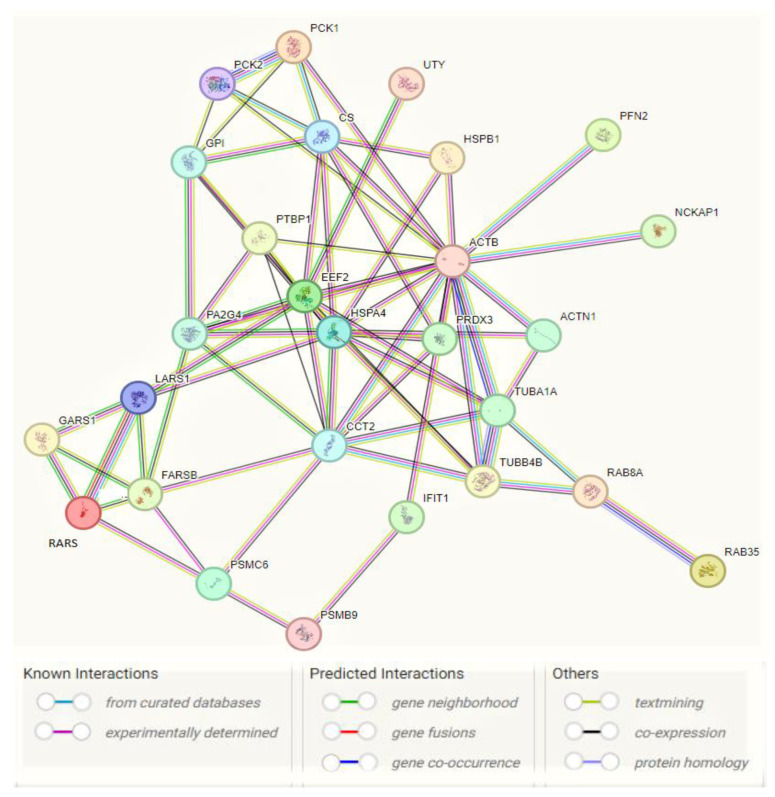
Interaction network of pro-tumorigenic (PT) proteins in MCF7 BC cell line transfected for JTB silencing, by means of STRING on-line database (https://string-db.org/, accessed on 22 October 2023). A total of 27 nodes and 69 edges were mapped in the PPI network with a PPI enrichment *p*-value of 6.44 × 10^−12^.

Cancer cells utilize glutamine metabolism for energy generation as well as to synthesize molecules that are essential for cancer growth and progression [141], such as nucleotides and fatty acids, which regulate redox balance in cancer cells [142]. PEPCKs increase the synthesis of ribose from non-carbohydrate sources, such as glutamine [39] as well as the serine and other amino acid synthesis [143]. Also, PCK1 helps regulate triglyceride/fatty acid cycle (GO_BP Regulation of lipid biosynthetic process) and development of insulin resistance (GO_BP Cellular response to insulin stimulus), being involved in glyceroneogenesis (GO_BP Glycerol biosynthetic pathway from pyruvate) and re-esterification of free fatty acids [144]. PEPCK-M is reported as a key mediator for the synthesis of glycerol phosphate from non-carbohydrate precursors, being important to maintain level of glycerophospholipids as major constituents of bio-membranes [145]. The effects of PEPCK on glucose metabolism and cancer cell proliferation are partially mediated by activation of mechanistic target of rapamycin (mTORC1) [39], which is regulated by glucose, growth factors and amino acids and is coupled to the insulin/IGF-1 (insulin-like growth factor 1) signaling pathway [146]. Thus, the mitochondrial phosphoenolpyruvate carboxykinase (PEPCK-M/PCK2), known to enhance cell proliferation and response to stress or nutrient/glucose restriction/deprivation in cancer cells (GO_BP Response to starvation) compared to PCK1 that functions primarily in gluconeogenesis, promotes tumor growth in ER+ BC through regulation of mTOR pathway (GO_BP Positive regulation of mTOR signaling) [40]. AMP-activated protein kinase (AMPK) is an ”energy sensor”/metabolic regulator involved in lipogenesis, glycolysis, TCA cycle, cell cycle progression, and mitochondrial dynamics [147]. PCK1 dysregulation may promote cell proliferation via inactivation of AMPK (KEGG_AMPK signaling pathway), known as a tumor suppressor [36] but was recently reconsidered as a putative oncogene [148]. PCK1-directed glycogen metabolic program regulates differentiation and maintenance of CD8^+^ T cells that are essential for protective immunity against cancer [139] (GO_BP Positive regulation of memory T cell differentiation). PEPCK is known to be activated in response to acidosis. The acid-induced PEPCK provides glucose for acid-base homeostasis (GO_BP Positive regulation of transcription from RNA polymerase II promoter in response to acidic pH) [149]. In conclusion, PCK enzymes are involved in gluconeogenesis, glyceroneogenesis, serine biosynthesis, and amino acid metabolism, targeting the increase of glucose level that contributes to the development and progression of many types of cancer arising in non-gluconeogenic tissues/organs [150]. 25 upregulated DEPs with PT activity (HSPB1, HSPA4, ACTB, ACTN1, PFN2, EEF2, PCK1, PCK2, FARSB, GARS1, LARS1, RARS1, PSMC6, CCT2, IFIT1, PTBP1, GPI, TUBA1A, UTY, CS, PA2G4, NCKAP1, PRDX3, RAB8A, and RAB35) and two downregulated DEPs (TUBB4B and PSMB9) were submitted for PPI network construction with STRING database (https://string-db.org/, accessed on 22 October 2023), to highlight the specific interaction network associated with the JTB^low^ condition in transfected MCF7 BC cell line (Figure 5). This enrichment indicates that these proteins with PT potential are biological connected, as a group.

**Figure 6:**
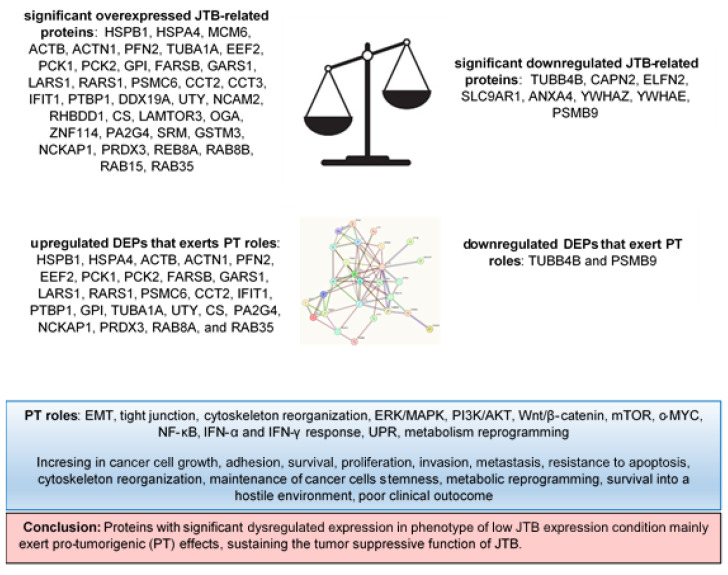
DEPs and their pro-tumorigenic (PT) activity in MCF7 BC cell line transfected for JTB silencing.

The main results of this experiment are synthetized in the Figure 6.

## 3. Materials and Methods

MCF7 cell culture, the transfection of hJTB plasmids and the collection of cell lysates was described previously [12] and briefly described below. 

### 3.1. Cell Culture

MCF7 cell lines were purchased from American Type Culture Collection (HTB-22 ATCC) and grown in RPMI medium supplemented with 10% FBS, 0.2% Gentamicin, 1% Penicillin-Streptomycin and 0.2% Amphotericin (growth media) at 37 °C. The cells were grown until they reached 70–80% confluency and were transiently transfected with JTB shRNA plasmid for downregulation.

### 3.2. Plasmids for Downregulation

Four plasmids were custom made by Creative Biogene, Shirley, NY, USA. Three shRNA plasmids containing GCTTTGATGGAACAACGCTTA sequence, with forward sequencing primer of 5′-CCGACAACCACTACCTGA-3′ and reverse primer of 5’-CTCTACAAATGTGGTATGGC-3′, GCAAATCGAGTCCATATAGCT sequence, with forward primer 5′-CCGACAACCACTACCTGA-3′ and reverse primer of 5′-CTCTACAAATGTGGTATGGC-3′, and GTGCAGGAAGAGAAGCTGTCA sequence with 5′-CCGACAACCACTACCTGA-3′ and reverse primer of 5′-CTCTACAAATGTGGTATGGC-3’, all targeting the hJTB mRNA respectively. The fourth plasmid was a control plasmid with a scramble sequence GCTTCGCGCCGTAGTCTTA with forward primer 5′-CCGACAACCACTACCTGA-3′ and reverse primer of 5′-CTCTACAAATGTGGTATGGC-3′. These plasmids were further customized to have an eGFP tag with Puromicin antibiotic resistance gene.

### 3.3. Transfection into MCF7 Cells

As stated in [12], Lipofectamine™ 3000/DNA and DNA/Plasmid (10 µg/µL) complexes were prepared in Opti-MEM Reduced Serum Media (Invitrogen, Waltham, MA, USA) for each condition and added directly to the cells in culture medium. Cells were allowed to grow for 48–72 h after which they were collected. 70% transient transfection efficiency was confirmed by visualizing the green fluorescence emitted by the eGFP using a confocal microscope (Figure S1).

### 3.4. Western Blot Analysis

Cell lysates from both the control and downregulated JTB condition were collected using a lysis buffer. The lysates were then incubated on ice for 30 min and centrifuged at 14,000 rpm for 20 min. The protein samples were quantified using Bradford Assay. Lysates containing 20 µg of proteins were run in a 14% SDS-polyacrylamide gels and transferred to nitrocellulose membranes. The blots were incubated with blocking buffer containing 5% milk and 0.1% tween-20 overnight at 4 °C with shaking. Primary antibody (JTB Polyclonal Antibody—PA5-52307, Invitrogen, Waltham, MA, USA) was added and incubated for 1 h with constant shaking. Secondary antibody (mouse anti-rabbit IgG-HRP sc-2357, Santa Cruz Biotechnology, Inc., Dallas TX, USA) was added and incubated for 1 h with constant shaking. After each incubation, the blots were washed thrice with TBS-T (1X TBS buffer, containing 0.05% tween-20) for 10 min each with constant shaking. Finally, the enhanced chemi-luminescent substrate (Pierce™ ECL Western Blotting Substrate—32106, ThermoFisher, Waltham, MA, USA) was added and the blot was analyzed using a CCD Imager. For normalization, mouse GAPDH monoclonal antibody (51332, cell-signaling technology, Danvers, MA, USA) was added and incubated for 1 h, followed by the addition of goat anti-mouse IgG-HRP (sc-2005, Santa Cruz Biotechnology) and the addition of ECL substrate. Image J software was used for the detection and comparison of the intensity of the bands (Figure S2).

### 3.5. 2D-PAGE & Proteomic Analysis

We used three biological replicates for the downregulated JTB condition. Two controls were used for the comparison: control (*n* = 3), control_shRNA (*n* = 3) and sh_JTB (*n* = 3). These conditions were analyzed in 2D-PAGE by Kendrick Labs, Inc. (Madison, WI, USA) and nanoLC-MS/MS as previously described [13]. The computer comparison was done for the average of three samples (3 vs. 3)—control_shRNA vs. sh_JTB (*n* = 3) and Control vs. sh_JTB (*n* = 3). The dysregulated spots were selected based on the criteria of having a fold increase or decrease of ≥1.7 and *p* value of ≤0.05. The data processing was done using PLGS software (v. 2.4) to convert them to pkl files and Mascot Daemon software (v. 2.5.1) was used to identify the dysregulated proteins. Finally, Gene Set Enrichment Analysis (GSEA) analysis was done to identify the identify the dysregulated pathways as previously described [13].

### 3.6. Data Sharing

Mascot data will be provided upon request, according to Clarkson University Material Transfer Agreement. The mass spectrometry data have been deposited to the ProteomeXchange Consortium via PRIDE partner repository with the dataset identifier PXD046265.4.

## 4. Conclusions

The jumping translocation breakpoint (JTB) protein has been reported as a regulator of mitochondrial function, cell growth, cell death and apoptosis, as well as a protein involved in cytokinesis/cell cycle. Some authors detected JTB as an overexpressed gene/protein in several malignant tissues and cancer cell lines, including liver cancer, prostate cancer, and BC, showing that this gene may suffer from unbalanced jumping translocation that leads to aberrant products, highlighting that the JTB downregulation/silencing increases cancer cell motility, anti-apoptosis, and promotes genomic instability and tumorigenesis. We also showed that the upregulated expression of DEPs in the JTB^low^ condition, investigated by SDS-PAGE followed by nLC-MS/MS proteomics in transfected MCF7 BC cell line, may promote cancer cell viability, motility, proliferation, invasion, ability to survive into hostile environment, metabolic reprogramming, escaping of tumor cells from host immune control, leading to a more invasive phenotype for MCF7 cells. Several downregulated DEPs in the JTB^low^ condition also promoted the invasive phenotype of MCF7 cells, sustaining cell proliferation, migration, invasion, and tumorigenesis. A plethora of DEPs identified during JTB silencing by in-solution digestion followed by nLC-MS/MS have been complementary and completed the list of DEPs identified by SDS-PAGE proteomics [12]. In this last case especially, upregulated proteins emphasized pro-tumorigenic activities in downregulated JTB state [13].

Using 2D-PAGE coupled with nLC-MS/MS proteomics, the present study identified 45 significantly dysregulated proteins, of which 37 were upregulated and 8 downregulated, in MCF7 BC cell line transfected for downregulated JTB condition. HSPB1, HSPA4, MCM6, ACTB, ACTN1, PFN2, TUBA1A, EEF2, PCK1, PCK2, GPI, FARSB, GARS1, LARS1, RARS1, PSMC6, CCT2, CCT3, IFIT1, PTBP1, DDX19A, UTY, NCAM2, RHBDD1, CS, LAMTOR3, OGA, ZNF114, PA2G4, SRM, GSTM3, NCKAP1, PRDX3, REB8A, RAB8B, RAB15 and RAB35 have been overexpressed, while TUBB4B, CAPN2, ELFN2, SLC9AR1, ANXA4, YWHAZ, YWHAE, and PSMB9 proteins were found to be significantly downregulated. GSEA revealed four upregulated pathways, including proteins important for interferon alpha (IFN-α) response, interferon gamma (IFN-γ) response, Myc targets V1, and unfolded protein response (UPR). Two downregulated pathways comprised proteins involved in estrogen response late and estrogen response early pathways. Almost all DEPs identified in this experiment exert pro-tumorigenic effects in the JTB^low^ condition, sustaining the tumor suppressive function of JTB. Thus, the identified DEPs are involved in several signaling and metabolic pathways that exert pro-tumorigenic roles: EMT, ERK/MAPK, PI3K/AKT, Wnt/β-catenin, mTOR, C-MYC, NF-κB, IFN-γ and IFN-α response, UPR, and glycolysis/gluconeogenesis. These pathways sustain cancer cell growth, adhesion, survival, proliferation, invasion, metastasis, resistance to apoptosis, cytoskeleton reorganization, maintenance of stemness, metabolic reprogramming, survival in a hostile environment, and a poor clinical outcome. In conclusion, JTB silencing might increase the neoplastic phenotype and behavior of MCF7 BC cell line.

Analysis of upregulation of JTB was systematic, complementary and comprehensive: in-solution digestion, 1-D-PAGE and 2D-PAGE, followed by proteomics. Analysis of JTB silencing was also systematic, complementary and comprehensive: in-solution digestion and 1D-PAGE, and now 2D-PAGE, followed by proteomics. Additional, complementary or better methods can also be used, at the sample level, or at the instrumentation level. The current in-solution and gel-based analysis can be complemented, among others, by peptidomics analysis, phsosphoproteomics analysis, or analysis of stable and transient protein-protein interactions. At the instrumentation level, 2D-UPLC could be one option, and newer, more performant mass spectrometers could also be used. 

Overall, taking into account the opinion of other authors, as well as based on our own team’s in vitro studies, we suggest that JTB protein might be considered as a tumor biomarker for BC and should be studied as a target for BC therapy.

## Figures and Tables

**Table 1:** Significant up and downregulated pathways in the downregulated JTB condition in the MCF7 BC cell line, according to GSEA with FDR < 25%.

	Pathways	NES	FDR q-Val
Upregulated	INTERFERON_ALPHA_RESPONSE (IFN-α)	1.52	0.223
	INTERFERON_GAMMA_RESPONSE (IFN-γ)	1.47	0.165
	MYC_TARGETS_V1	1.04	0.971
	UNFOLDED_PROTEIN_RESPONSE	1	0.831
Downregulated	ESTROGEN_RESPONSE_LATE	−1.17	1
	ESTROGEN_RESPONSE_EARLY	−1.07	0.948

**Table 2:** Deregulated DEPs, neoplastic roles, and biological processes expressed in response to JTB downregulation in MCF7 BC cell line.

Gene Name	Gene Description	Role	Expression in Malignancies and Putative Neoplastic Effects		Pathways	Neoplastic Condition
Proteins enriched in phenotype of low JTB expression condition						
HSPB1/HSP27	Heat shock 27 kDa protein 1	protein folding/CMPF, cell adhesion, cell migration, cytoskeleton dependent intracellular transport, cell death in response to OS, programmed cell death	overexpressed in many cancers [17]: including BC [18]	PT	APOPTOSIS, regulates EMT process and NF-κB activity [18]	poor clinical outcome, cell invasion, metastasis, resistance to apoptosis [17]; participates in maintenance of BCSCs [18]
HSPA4/HSP70/Apg-2	Heat shock 70 kDa protein family A (HSP70) member 4 isoform a variant	molecular chaperone, induced by oncogenic stress, autophagy, regulation of protein ubiquitination, cadherin binding, cell adhesion molecule binding, MHC class II protein complex binding [19]	overexpressed in many cancers, including HCC [19], CRC [20], HNSC [21], involved in progression of BC [22], GC [23]	PT	MTORC1_SIGNALING; associated with CSCs proprieties via chaperones for EMT-associated proteins, inducing migration [23]; silencing reduced activation of PI3K/Akt signaling and increase in apoptosis [20]; activates mTOR pathway [21]	facilitates cancer cell survival, inhibits apoptosis, promotes proliferation, immune regulation [19], accumulation of misfolded proteins, ROS, and DNA damage [21], considered as a necroptosis-related gene in BC that activates cell cycle [22]
MCM6	Minichromosome maintenance complex component 6	significant DNA replication regulator, plays a key role in cell cycle progression [24]	overexpressed in many cancers [25]: BC, CRC, HCC [25], glioma, endometrial adenocarcinoma, cervical cancer, Merkel cell carcinoma, lung cancer [24]	PT	promotes EMT and activates MEK/ERK signaling, sustaining carcinogenesis [24,25]	sensitive, specific biomarker for GSTM cancer, involved in cell proliferation, metastasis, migration, invasion, immune response [24,25]
POTEF/POTEACTIN/ACTB	POTE ankyrin domain family member F/Beta-actin	cytoskeleton protein that belongs to the actin family, involved in motility, polarity, chemotaxis andimmune cell infiltration [26]	expressed in many cancers: BC cell lines, overexpressed in CRPC [27]	PT	AJ; represses the AT effect of Toll-like receptor (TLR) signaling pathway [27,28]; involved in cell migration by NF-κB and Wnt/β-catenin pathway [26]	promotes cell growth [27], migration, invasion [26]
ACTN1	Alpha-actinin-1 (isoform X5)	actin cross-linking protein involved in cytokinesis, cell adhesion and migration [29]	overexpressed in BC tissue, BL-BC cell lines [30], GC [29]	PT	AJ; EMT via AKT/GSK3β/β-catenin pathway and FAK/Src/JAK2/STAT3 signaling [29]	promotes cell proliferation, invasion, migration, and inhibits apoptosis [29]; loss of polarity by cytoskeleton reorganization and E-cadherin-based adhesion, lack of ER expression and poor survival in BL-BC [30]
PFN2	Profilin-2 (isoform b)	actin binding protein involved in cytoskeleton organization, vesicle mediated transport, signaling, cell junction organization, cell motility	overexpressed in TNBC [31], ESCC [32]	PT	EMT [31]	promotes cell proliferation, migration and invasion of TNBC cells [31]
EEF2	Eukaryotic elongation factor 2	translation elongation factor involved in polypeptide chain elongation step, cell cycle progression	highly expressed in various malignant tumors: BC [33], GC, CRC, esophageal, pancreatic, PCa, HNSCC, GBM [34], LSCC tissues and cell lines [35]	PT	UPR; promotes G2/M progression in cell cycle activating Akt signaling [34], CDC2/Cyclin B1 and EMT-related proteins [35]	associated with node positivity [33]; plays an oncogenic role, promotes cancer cell growth [34], migration, invasion [35]
1KHB/PCK1/PEPCK-C (cytoplasmic isoenzyme)	Phosphoenolpyruvate carboxykinase 1 (PEPCK) Complex With Nonhydrolyzable GTP Analog, Mad Data (chain A)	rate-limiting gluconeogenesis enzyme	oncogene overexpressed in colon cancer and melanoma [36]; downregulated and tumor suppressor in gluconeogenic tissues (liver and kidney): HCC [36] and ccRCC [37]	PT	GLYCOLYSIS; acts via AMPK/p27^Kip1^ axis [36]; depletion promotes EMT in HCC [38]	promotes cell proliferation via mTORC1 (oncogenic function) [39]; AT in kidney and liver: suppressed ccRCC cell growth and metastasis, inhibited tumorigenesis by blocking aerobic glycolysis pathway [37]; suppresses liver tumor growth, cell cycle progression and proliferation [36]
PCK2/PEPCK-M (mitochondrial isoenzyme)	Phosphoenolpyruvate carboxykinase	rate-limiting gluconeogenesis enzyme	overexpressed many cancers, including ER^+^ BC [40], lung, prostate, thyroid, bladder, BC, cervical cancer [41]	PT	REACTOME_GLUCONEOGENESIS; activation of mTORC1 and E2F1 pathways [40]; silencing contributes to cellular senescence, inhibiting EMT in BC cells [42]	promotes tumor growth, proliferation and cell cycle progression [40]
FARSB/HSPC173	Phenylalanyl-tRNA ligase beta subunit	cytoplasmic aminoacyl-tRNA synthetase (ARS/AARS) involved in tRNA metabolic process, amino acid metabolic process, protein-containing complex assembly	overexpressed in in tumor samples compared to adjacent normal tissues [43]: GC [44]	PT	aminoacyl-tRNA synthesis pathway [44]	promotes cancer progression, poor prognosis, metastasis [44]; worse patient survival in BC [43]
GARS1	Glycyl-tRNA synthetase 1	cytoplasmic and mitochondrial ARS	overexpressed in tumor samples compared to adjacent normal tissues [43], displays androgen-dependent transcriptional initiation in several hormone-responsive cells, overexpressed in PCa [45]	PT	may deactivate ERK signaling pathway [45]	worse patient survival in BC [43]; could induce tumor regression [45]
LARS1	Leucyl-tRNA synthetase 1 (Editing Domain)	cytoplasmic ARS	overexpressed in some cancers: myeloid leukemia, pancreatic cancer, renal, cervical, skin cancer [45], lung cancer cell lines and tissues [46]	PT	senses intracellular leucine levels to activate mTORC1 pathway [45]	lower patient survival in BC [43], promotes cell proliferation, growth [45], and migration [46]
RARS1	Arginyl--tRNA synthetase 1	cytoplasmic ARS involved in protein synthesis	important tumorigenic activity, overexpressed in hepatoma cells [47], associated with an increased risk of BC [45]	PT	impairment of AIMP1/EMAPII secretion in MCF7 cells [48]	lower patient survival in BC [43], increases growth rate in hepatoma cells, induces stem cell-like features in head and neck tumors [47]
PSMC6	Proteasome subunit p42/proteasome 26S subunit ATPase 6	protein catabolic process, regulation of DNA-template transcription, protein-containing complex assembly	highly upregulated in LUAD [49], BC tissues, especially in luminal cancer [50]	PT	MTORC1_SIGNALING; REACTOME_REGULATION_OF_MITOTIC_CELL_CYCLE; activation of Wnt signaling via degrading AXIN proteins [49]	oncogenic effect [50], poor prognosis, silencing inhibits cell growth, migration and invasion [49]
CCT2/CCTβ	Chaperonin containing TCP1 Subunit 2	involved in cell cycle regulation, protein folding and binding biological processes [51]	overexpressed in various tumors and cell lines, such as HER2+ BC, liver, prostate, cholecyst, lung, CRC, BC [51], glioblastoma [52]	PT	P53 signaling [51]	worse prognosis, especially in luminal A subtype, promotes cell growth/survival, invasion and proliferation [51]
CCT3/TRiC	Cytosolic chaperonin containing t-complex polypeptide 1 (TCP1) subunit 3/TCP1 ring complex (hTRiC5), partial	molecular chaperone involved in proteostasis, folding of tubulin and actins and many proteins involved in cancer [53], cell division, proliferation andapoptosis [52]	overexpressed in some tumors: BC, HCC, LUAD and LUSC, NSCLC, cervical and CRC, AML, multiple myeloma, papillary thyroid carcinoma, melanoma, GC [52]	PT	might regulate IGF-1 signaling; actin cytoskeletal signaling, and PTEN signaling, Wnt/β-catenin, JAK2/STAT3, PI3K/Akt [52]	oncogene, promotes cell growth, survival, proliferation, cell cycle progression and anti-apoptosis [52]
IFIT1	Interferon-induced protein with tetratricopeptide repeats 1 (isoform 2)	inflammation-related protein, RNA-binding protein modulated by JAK/STAT pathway [54], involved in regulation of translation [55]	overexpressed in PDAC [54], OSCC [56]	PT	INTERFERON_GAMM_RESPONSE; UPR; EMT [56], Wnt/β-catenin activation [54], increasing levels of p-EGFR and p-Akt [56]	increases cell proliferation, migration, invasion [54], tumor growth, regional and distant metastasis [56]
PTBP1/hnRNP1	Polypyrimidine tract-binding protein 1 (isoform a)	RNA-binding protein, key factor in the control of RNA metabolism, regulates mRNA alternative splicing (AS) events, mRNA stability, mRNA localization [57]	overexpressed in human epithelial ovarian tumors, BC tissues and cell lines, glioblastomas [57], LUAD tissues and cell lines [58]	PT	regulates PTEN-PI3K/Akt and autophagy [57]; enhances EGFR signaling, MAPK, hypoxia inducible factor-1α pathways [59]; associated with HSP progress [58]	associated with breast tumorigenesis, promotes tumor cell growth, invasion and maintenance of metastasis [57], regulates apoptosis and cell proliferation [59]
DDX19A	DEAD-box helicase 19A/ATP-dependent RNA helicase DDX19A	RNA helicases involved in RNA metabolic process including transcription, RNA transport, RNA degradation [60]	overexpressed in CSCC [60], BC cell lines [61]	PT	induces EMT [60]	promotes cell migration, invasion, metastasis, NOX1 expression and ROS production [60]
GPI	Glucose-6-phosphate isomerase/neuroleukin	cytoplasmic glycolytic-related enzyme, secreted in ECM of cancer cells it is called autocrine motility factor (AMF) [62] and functions as a cytokine or growth factor [63]	overexpressed in BC [63], LUAD/NSCLC, glioblastoma, ccRCC [64], GC [62]	PT	REACTOME_GLUCONEOGENESIS; OXPHOS; glycolysis and gluconeogenesis [64], correlated with cell cycle regulatory genes, immune cell infiltration, gene alteration, ferroptosis genes [63]; AMF induces EMT [65]	involved in cell cycle, cell proliferation, correlates with immune infiltration, cell migration, invasion [64]; silencing suppressed proliferation, migration, invasion, glycolysis, and induced apoptosis [62]
TUBA1A	Tubulin alpha-I a	cell division, cell movement, microtubule based process, cell junction organization, cytoskeleton organization and cytoskeleton dependent intracellular transport	upregulated in BC tissues [66], GC [67]	PT	involved in infiltration of macrophages to the tumor microenvironment [67], involved in EMT related to re-organization of cell-cell contact [68]	overexpression was correlated with poor overall survival and a more aggressive phenotype in GC [67]
UTY/KDM6C	Ubiquitously transcribed tetratricopeptide repeat protein Y-linked transcript variant 83	member of lysine (K)-specific dimethylase (KDM6) family that act as dynamic regulators of gene expression by histone demethylation [69], chromatin organization, anatomical structure development, gene transcription regulation [70]	not	controversial	REACTOME_CHROMATIN_MODIFYING_ENZYMES, transcriptional dysregulation in cancer (KEGG)	oncogenic or tumor suppressive roles [70]
NCAM2	Neural cell adhesion molecule 2	involved in cell adhesion, differentiation and anatomical structure development	overexpressed in some prostate and BC cell lines, such as ER-dependent BC cell lines MCF7 and T47D [71]	not known	not known	not known
RHBDD1	Rhomboid-related protein 4 isoform X1/rhomboid domain-containing protein 1	intramembrane/cytoplasmic-cleaving serine protease involved in intracellular protein transport, programmed cell death, protein catabolic process, cell differentiation, cell growth, protein maturation, participates in ER quality control system [72] and regulation of mitochondrial membrane remodeling [73]	highly upregulated in BC, CRC tissue and cell lines [72]	PT	positive correlation with p-Akt and CDK2 [72], c-Jun [73]	important in tumorigenesis, poor prognosis in ER+, ER+PR+, HER2+, and TNBC, inhibits apoptosis by activation of c-Jun and Bcl-3 [73]; deletion suppresses BC cell survival, migration, invasion, cycle progression and G1/S phase transition and increases apoptosis and ERAD [72]
CS	Citrate synthase	rate-limiting respiratory enzyme in the TCA cycle, involved in cell lipid metabolism (conversion of glucose to lipids) and mitochondrial function [74]	overexpressed in various cancers: PCa [74], pancreatic ductal carcinoma [75], ovarian carcinoma [76]; downregulated in human cervical carcinoma cells [77]	PT	OXPHOS; lipid metabolism signaling [74]; downregulation could induce EMT [77]	aggressive tumor progression, poor prognosis, increases cell proliferation, growth, colony formation, migration, invasion, and cell cycle [74]; silencing induces reduction of cell proliferation, invasion, migration, and enhances apoptosis [76], cells being unable to grow or proliferate in response to extracellular growth factors [78]
LAMTOR3/MAPKSP1/MP1	Late endosomal/lysosomal adaptor, MAPK and MTOR activator 3/mitogen activated protein kinase scaffold protein 1/MEK partner 1	member of the Ragulator complex involved in multiple signaling pathways that acts as a scaffold protein complex [79]	overexpressed in ER+ and ER- BC cell lines and in non-tumorigenic mammary epithelial cell lines [80]; downregulated in KIRC [79]	PT	considered to be a convergence point for MAPK and mTOR pathways [81]; targeting MEK1/MP1/ERK1/BCL2 axis may improve clinical outcome of MLCC patients [82]	required for pro-survival signaling from PI3K/AKT pathway in ER+ BC cells [80]; upregulation induces BCL2 expression (anti-apoptotic protein) [82]
OGA	Protein O-GlcNAcase (isoform a)	involved in protein glycosylation and protein catabolic process	overexpressed in numerous cancers [83]	PT	drives aerobic glycolysis and tumor growth by inhibiting PKM2 [83]	enhances tumor progression [83];
ZNF114	ZNF114 protein, partial	DNA-binding protein involved in transcription; member of KRAB-ZEPs family of transcription regulators [84]	overexpressed in ccRCC [85]	PT	pluripotency maintenance; repression of differentiation gene DPYSL4 [86]	involved in maintenance of cell pluripotency and stemness [84,86]; shorter overall survival [85]
PA2G4/EBP1	Proliferation associated protein 2G4/ErbB3-binding protein 1	found in cytoplasm and nucleus, regulates cell growth and differentiation, being a ribosomal constituent, transcriptional regulator, RNA/DNA-binding protein, mediates rRNA processing, DNA transcription, mRNA translation, protein stability and signal transduction [87]	overexpressed in HCC, cervical cancer, CC, NPC, salivary ACC, downregulated in HER2+ BC and bladder cancer [87]	PT	tumor formation via Ebp1/p38/HIF1α signaling and proto-oncogene MDM2-mediated downregulation of p53, promotes EMT [87]	intensively involved in tumorigenesis and cancer progression/metastasis, promotes cell proliferation and soft agar colony generation [87]
SRM	spermidine synthase	essential polyamine for cell proliferation, differentiation, development [88], regulation of gene expression, apoptosis, cell cycle progression and signaling pathways [89]	biosynthesis is upregulated in BC and contribute to disease progression [89], overexpression in CRC [90]	PT	MYC_TARGETS_V2; interferes with mTOR and RAS oncogenic pathways [88], c-MYC target [91] and C/ERPβ may serve as regulators of SRM [90]	cell growth [89]
GSTM3	Glutathione S-transferase mu3	enzyme involved in xenobiotic metabolism/detoxification, apoptosis inhibition [92], regulates ROS and participates in OS-mediated pathology [93]	mRNA expression level high in HER2+ or ER+ BC [93], overexpressed in cervical cancer, colon cancer [92]	PT	EMT inducer [94], cellular stress response via NF-κB and MAPK pathway during tumor progression [92]	cancer cell maintenance, survival and tumor progression [92]
NCKAP1/NAP1	Noncatalytic region of tyrosine kinase (Nck)-associated protein 1	associates with Src homology 3 (SH3) domain of NCK protein that localizes along the lamellipodia and mediates contact-cell dependent migration [95], member of WASF3 regulatory complex [96]; involved in cytoskeleton organization through actin polymerization, programmed cell death, signaling, cell differentiation and protein-containing complex assembly	overexpressed in NSCLC [95]; downregulated in ccRCC [97]	PT	ribosomal signaling, OXPHOS, TGF-β, EMT-related signaling pathways [97]; involved in HSP90-mediated invasion and metastasis by provoking MMP9 activation and EMT [95]	overexpression is associated with poorer survival in BC patients; essential for cell motility, adhesion, invasion and metastasis [95]; knockdown in BC cell lines, MDA-MB-231 and Hs578T, leads to a significant reduction in invasion and suppresses metastasis [96]; tumor suppressive in ccRCC [97] and HCC [98]
PRDX3	Thioredoxin-dependent peroxide reductase, mitochondrial isoform a precursor	mitochondrial member of the antioxidant family of thioredoxin peroxidase [99] required for mitochondrial homeostasis [100]	overexpressed in HCC, malignant mesothelioma, BC, PCa, lung cancer, cervical carcinoma [99]; overexpression associated with ER and PR [100]	PT	OXPHOS; defense against H2O2 produced by mitochondrial respiratory chain [99], c-Myc target gene [100]	tumor promoting effects [99], involved in regulation of cell proliferation, differentiation and antioxidant function, overexpression protects cells from oxidative stress and apoptosis [100]
RAB8A	Ras-related protein Rab-8A	intracellular protein transport, intracellular membrane trafficking, autophagy, vesicle-mediated transport, signaling, cell differentiation, membrane organization	overexpressed in BC tissues [101]	PT	activates AKT and ERK1/2 signaling pathways [101]	increases cell growth, proliferation, migration, invasion [101]
RAB8B	Ras-related protein Rab-8B	member of the Rab small G protein family, immune system process, intracellular trafficking, peroxisome organization, vesicle-mediated transport, signaling, cell junction organization, membrane organization, protein localization to plasma membrane	overexpressed in TC [102]	PT	required for Wnt/β-catenin signaling [103]	overexpression and loss of functioning adherence junction accelerate tumorigenesis in testis [102]
RAB15	Ras-related protein Rab-15 (isoform AN2)	involved in trafficking of cargos through the apical recycling endosome (ARE) to mediate transcytosis [104]	overexpressed in liver cancer cells [105]	PT	regulates the endocytic recycling pathway [106]	associated with the susceptibility of cells to DNA damage-induced cell death [105]
RAB35	Ras-related protein Rab-35 (isoform 1)	Rab GTPase located in plasma membrane/endosomes, involved in vesicular trafficking, actin dynamics, cytokinesis, apical-basal polarity, endocytosis, phagocytosis, autophagy, exosome release [107]	overexpressed in OC [107]	PT	activator of PI3K/AKT pathway [107]	oncogenic protein, enhances BC cells invasion and metastasis [107]
Proteins with downregulated expression in phenotype of low JTB expression condition						
TUBB4B	tubulin beta-4B chain	constituent of microtubules involved in mitotic cycle, immune system process and cytoskeleton organization	overexpressed in membranes of stem cells enriched cultures, PCa, OC, glioblastoma, metastatic CRC [108]	PT	downregulation is essential for the initiation of EMT [108], for microtubule-VIM interaction and contributes to the maintenance of polarity in migrating cells [108]	decreased level correlates with increased cell migration [109]
CAPN2	Calpain 2/m-calpain	calcium-dependent, non-lysosomal cysteine protease involved in lipid metabolic process, cytoskeleton organization, programmed cell death, cell differentiation, protein catabolic process, protein maturation and anatomical structure development	overexpressed in various malignancies: CRPC cell lines (DU145, PC3), BL and TNBC [110], RCC [111], PC [112]	AT	acts via AKT/mTOR signaling pathway [111], and regulates Wnt/β-catenin signaling pathway-mediated EMT [112]; silencing may inhibit EMT [111,112]	oncogene involved in carcinogenesis and tumor progression, and metastasis silencing inhibits cell proliferation, migration and invasion by reducing MMP-2 and MMP-9 activation and regulation of invadopodia dynamics [111]
ELFN2/LRRC62	Extracellular leucine rich repeat and fibronectin type III domain-containing 2/protein phosphatase 1 regulatory subunit 29	putative oncogene, hypomethylation gene [113]	overexpressed in GC tissues and cell lines [114]; oncogene in GBM [113]	AT	interacts with AurkA and eukaryotic translation initiation factor 2 subunit alpha (EIF2α) and regulates the kinase activity of AurkA to promote cell autophagy [113]	knockdown inhibits cell proliferation, migration, invasion, increases E-cadherin and decreases N-cadherin [114]
SLC9AR1/NHERF1/ERB50	Solute carrier (SLC) family 9 (Na(+)/H(+) exchanger), member 3 regulator 1/sodium-hydrogen exchanger regulatory factor 1/ERM-binding phosphoprotein	multifunctional cytoplasmic adaptor involved in growth factor signaling [115]; interacts with several proteins related to the estrogen pathway and tumorigenesis: EGFR, PTEN, PDGFR, beta catenin, EZR [116]	in BC acts as a tumor suppressor protein; oncogene in glioma and other cancers; overexpressed in PCa tissue and cell lines [117]	AT/PT	MTORC1_SIGNALING; ESTROGEN_RESPONSE_LATE; downregulation is associated with Wnt/β-catenin inactivation [118]; knockdown enhances PDGF-induced cytoskeletal rearrangements and chemotactic migration of cells [119]; AKT-associated protein [115]	knockdown suppresses proliferation and migration of metastatic PCa cells and promotes apoptosis [117]
ANXA4	Annexin 4/lipocortin IV/endonexin I [120]	intracellular Ca2+ sensor that modulates membrane permeability and membrane trafficking, participates in cell growth, apoptosis [121], cell cycle and anticoagulation [122]	overexpressed in various tumors: chemoresistant LC, ESCC, GC, CRC, PCC, gallbladder, HCC, cholangiocarcinoma, BC, RCC, OCCC, laryngeal and PCa, MM [121,123], cervical cancer [120]	AT	overexpressed, is related to AKT, CDK1, and tumor suppressor p21 [122]	knockdown attenuates migration in OC and BC cells [121]
YWHAE	14-3-3 protein epsilon	mitochondrial import stimulation factor L subunit [124] involved in mitotic cell cycle, intracellular protein transport, nucleocytoplasmic transport, signaling, cell differentiation, cell motility and transmembrane transport	overexpressed in BC tissue [124]	AT	MITOTIC_SPINDLE; MYC_TARGETS_V1	knockdown reduces expression of Snail and Twist [124]
YWHAZ	tyrosine 3-monooxygenase/tryptophan 5-monooxygenase activation protein zeta	central hub protein involved in many signal transduction pathways [125]	oncogene overexpressed in multiple cancers: HCC, CRC, LUAD, BC [125], urothelial carcinomas [126]	AT	UPR	knockdown decreases cell growth, proliferation, invasion, enhances apoptosis and tamoxifen-induced inhibition of cell viability [125]
PSMB9/LMP2	Proteasome 20S subunit 9 beta/low molecular weight protein 2	immunoproteasome functions; major enzyme in ubiquitin-dependent protein degradation and inactivation [127]	overexpressed in tumor tissues: LGG [127]; deficient in uLMS [128]	PT	INTERFERON_ALPHA_RESPONSE; INTERFERON_GAMMA_RESPONSE; downregulation is associated with inhibition of pathways related to formation and development of ECM through proto-oncogene tyrosine-protein kinase (SRC) gene	defective expression contributes to abnormal cell proliferation and tumor progression [128]

**Abbreviations:** ACC—adenoid cystic carcinoma; AJ—APICAL_JUNCTION; AT—anti-tumorigenic; BC—breast cancer; BCSCs—breast cancer stem cells; CC—colon cancer; ccRCC/KIRC—clear cell renal cell carcinoma/kidney renal clear cell carcinoma; CDK2—cyclin-dependent kinase 2; CMPF—chaperone-mediated proteins folding; CRC—colorectal cancer; CRPC— castration-resistant prostate cancer; CSCC—cervical squamous cell carcinoma; DCIS—ductal carcinoma in situ; EMT—epithelial–mesenchymal transition pathway; ERAD—endoplasmic reticulum-associated degradation; ERK—extracellular signal-regulated kinase; ESCC—esophageal squamous cell carcinoma; EZRezrin; FAK—focal adhesion kinase; GBM—glioblastoma multiforme; GC—gastric cancer; HCC—hepatocellular carcinoma; HNSC—head and neck squamous carcinoma; IAP—inhibitory of apoptosis proteins; KRAB-ZEPs—Krűppel-associated box domain zinc finger proteins; LC— lung cancer; LGG—low grade glioma; LSCC—lung squamous cell carcinoma; LUAD—lung adenocarcinoma; LUSC—lung squamous cell carcinoma; MAPK—mitogen-activated protein kinase; MDM2—mouse double minute 2 homolog/E3 ubiquitin-protein ligase; MM—malignant mesothelioma; MMP9—metalloproteinase 9; mTOR—mammalian target of rapamycin; NOX1—NADPH oxidase 1; NPC—nasopharyngeal carcinoma; NSCLC—non-small-celllung cancer; OCCC—ovarian clear cell carcinoma; OSCC—oral squamous cell carcinoma; OXPHOS—oxidative phosphorylation pathway; PCC—pancreatic cancer; PCa—prostate cancer; PDAC—pancreatic ductal adenocarcinoma; PT—pro-tumorigenic; PTEN—phosphatase and tensin homolog; ROS—reactive oxygen species pathway; TC—testicular cancer; TCA—tricarboxylic acid; TGF-β—transforming growth factor beta; uLMS—uterine leiomyosarcoma; UPR—unfolded protein response; VIM—vimentin.

## Data Availability

The mass spectrometry data have been deposited to the ProteomeXchange Consortium via PRIDE partner repository with the dataset identifier PXD046265.

## Supplementary Materials

The following supporting information can be downloaded at: https://www.mdpi.com/article/10.3390/molecules28227501/s1, Figure S1. Confocal microscope images showing conformation of transient transfection for control (A) and JTB downregulated condition (B). Left panel is the Bright Field (BF) mode, middle panel is the GFP mode and the right panel is a merge between BF and GFP modes. Figure S2. Downregulation confirmation of hJTB compared to control samples with (A) showing the downregulation of JTB protein at ~45 kDa in MCF7 cells treated with sh plasmids compared to control using commercially available full length hJTB antibody from Invitrogen; (B) shows GAPDH used as the loading control at 37 kDa.
