# Supplementary material for: Two-Dimensional-PAGE Coupled with nLC-MS/MS-Based Identification of Differentially Expressed Proteins and Tumorigenic Pathways in MCF7 Breast Cancer Cells Transfected for JTB Protein Silencing
Source: Molecules. 2023 Nov 9;28(22):7501. doi: 10.3390/molecules28227501 (PMC10673289; doi:10.3390/molecules28227501)
Supplement: Supplementary file 1 [file molecules-28-07501-s001.zip › molecules-2662242-supplementary.pdf]

Supplementary Materials

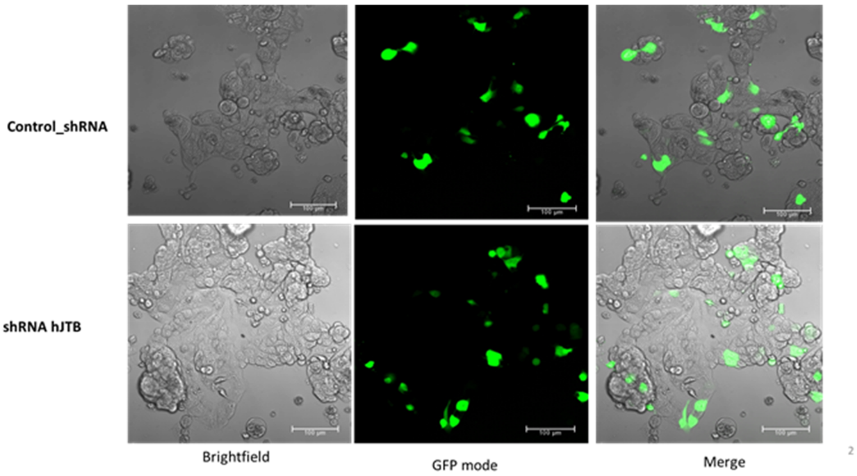

Figure S1. Confocal microscope images showing conformation of transient transfection for control (A) and JTB downregulated condition (B). Left panel is the Bright Field (BF) mode, middle panel is the GFP mode and the right panel is a merge between BF and GFP modes [1].

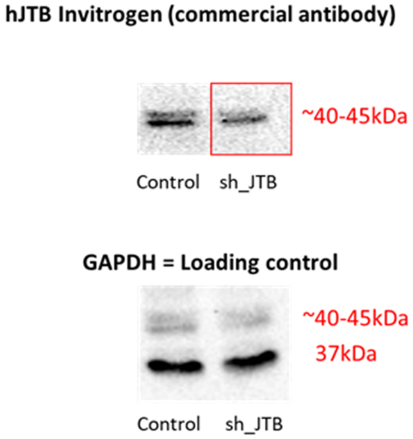

Figure S2. Downregulation confirmation of hJTB compared to control samples with (A) showing the downregulation of JTB protein at ~45 kDa in MCF7 cells treated with sh plasmids compared to control using commercially available full length hJTB antibody from Invitrogen; (B) shows GAPDH used as the loading control at 37 kDa [1].

References

1. Jayathirtha, M., et al., *Investigation of the effects of downregulation of jumping translocation breakpoint (JTB) protein expression in MCF7 cells for potential use as a biomarker in breast cancer.* American journal of cancer research, 2022. **12**(9): p. 4373-4398.
